# Impact of Anesthesia Strategy on Infant Pulmonary Function Test Quality and Duration

**DOI:** 10.1002/ppul.71477

**Published:** 2026-01-22

**Authors:** Aditi K. Zaveri, Brian Yoho, Brian Blasiole, Erick Forno, Daniel J. Weiner, Kristina Gaietto

**Affiliations:** ^1^ Division of Pulmonary Medicine, Department of Pediatrics UPMC Children's Hospital of Pittsburgh Pittsburgh PA USA; ^2^ Department of Anesthesia and Perioperative Medicine UPMC Children's Hospital of Pittsburgh Pittsburgh PA USA; ^3^ Division of Pulmonology, Allergy/Immunology, and Sleep Medicine, Department of Pediatrics Riley Hospital for Children Indianapolis IN USA

**Keywords:** infants, pediatric anesthesia, pulmonary function test

## Abstract

**Introduction:**

While chloral hydrate (CH) has been standard for infant pulmonary function testing (iPFT) sedation, CH shortages are necessitating use of different sedation approaches. We aimed to compare the safety, test duration, and test quality of alternative sedation strategies for iPFT.

**Methods:**

We conducted a retrospective chart review of iPFT conducted at our center from January 2019 to December 2021. We manually abstracted patient demographics, sedation medications given, adverse events, and iPFT type (raised volume‐rapid thoracic compression, plethysmography, bronchodilator response, and/or multiple breath washout), duration (induction, procedure, recovery, and total times), and quality (satisfactory vs unsatisfactory), then compared features of tests conducted with CH to tests conducted with ketamine and midazolam (KM), dexmedetomidine (DX), or multiple agents (polypharmacy, PP) using bivariate and multivariable analysis.

**Results:**

Sixty‐six children had iPFT (CH *n* = 42, KM *n* = 10, PP *n* = 8, and DX *n* = 6). Testing types and proportion of satisfactory tests did not significantly differ between CH and the other sedation strategies. In the multivariable analysis, compared to CH, we found that procedure time was shorter for KM, induction time was shorter for DX, and recovery time was longer for DX, yet total testing duration did not differ between CH and KM (*p* = 0.61) or DX (*p* = 0.22). In adjusted analyses, total testing time was longer for PP compared to CH (*β* = 12.0 min, *p* = 0.047). Adverse events, all of which were mild, occurred in three patients (PP *n* = 2, DX *n* = 1).

**Conclusions:**

Our findings provide preliminary evidence that KM, DX, and PP may be safe and effective alternatives to CH for iPFT sedation.

## 1 Introduction

Infant pulmonary function testing (iPFT) provides physiologic information early in the course of pediatric lung disease. iPFT encompasses a variety of tests, including tidal breathing measures, rapid thoracic compression spirometry, raised‐volume thoracic compression spirometry and plethysmography. While older children can reliably perform breathing maneuvers required for lung function testing, most children younger than 3 years are unable to perform the forced exhalation maneuvers required for spirometry. iPFT addresses this challenge with the use of a pneumatic vest and face mask to mimic forced inspiratory and expiratory maneuvers while under sedation. Optimal sedation can be challenging for this procedure, given that it requires the infant to be still enough to tolerate a variety of stimulating maneuvers while maintaining a patent airway and spontaneous breathing.

Since the initial development of iPFT in the 1980s, chloral hydrate (CH) has been the most used sedative agent [1, 2]. It is an orally administered drug with minimal effects on respiratory drive and airway tone when administered at typical doses. It does not require intravenous access nor the presence of an anesthesiologist [3]. Additionally, iPFT published normative data have all been collected under the use of CH [4, 5]. However, availability of CH in the USA has been limited since the discontinuation of commercially available CH syrup products in 2012 by Pharmaceutical Associates and Breckenridge due to business reasons [6, 7]. Some hospital pharmacies would compound the oral syrup from chloral hydrate crystals/powder, which unfortunately have also had limited availability. Additionally, several reports described an increase in the rates of sedation failure or prolonged sedation when using the compounded formulation [8, 9].

Given the frequent unavailability of CH and the risks associated with undersedation using the compounded variety, alternative sedation strategies have been utilized with more frequency and experience within our institution. These strategies have included some combination of ketamine, dexmedetomidine, midazolam, or propofol administered via the intranasal, intramuscular and intravenous routes. Each of these medications –alone or in combination– carries a unique risk/benefit profile when it comes to effectiveness in completing iPFT.

Currently, there is a paucity of data describing the efficacy of alternative sedation approaches for iPFT. The aim of this study was to retrospectively evaluate the impact of different sedation strategies on the procedure times and quality of iPFT.

## 2 Methods

This study was approved by the Institutional Review Board of the University of Pittsburgh (STUDY22010021) with a waiver of informed consent, as there were no research or study procedures (i.e., all data were retrospectively abstracted from the electronic health record); recognized standards have been followed. We conducted a retrospective chart review of all iPFT conducted at our center between January 2019 and December 2021. iPFT was performed with the Infant Pulmonary Lab (nSpire) and included tidal breathing measures, plethysmography, and raised‐volume rapid thoracic compression (RV‐RTC). Tidal breathing measures, plethysmography, and RV‐RTC were obtained for all infants in the study for clinical purposes. Bronchodilator responsiveness (BDR) was also typically conducted as part of an infant's first iPFT for clinical purposes. SF6 multiple breath washout (MBW) was sometimes added as an additional test for a separate multicenter clinical research study using a proprietary research instrument, an Innocor device modified by removing the pneumotach and using separate wash‐in and washout bags [10], designed for the Longitudinal Infant Multi‐Breath washout Study (LIMBuS). Acceptability criteria for the iPFT and BDR protocol in this study [10, 11, 12, 13] are detailed in the Online Supplement Methods. One pediatric pulmonologist and one respiratory therapist conducted all infant pulmonary function tests included in this study. CH was administered by the pediatric pulmonologist, and all other sedation medications were administered by a pediatric anesthesiologist or CRNA.

We reviewed anesthesia records for each study to abstract sedative drug(s) including dosages and the sequence of drugs administered for the procedure. Sedation strategies included CH, Ketamine and Midazolam (KM), Dexmedetomidine (DX), or a combination of more than two drugs, referred to as polypharmacy (PP). CH was given orally, and Ketamine and DX were given intravenously. Midazolam was generally given intranasally, but if a patient had an intravenous line they may have received doses intravenously. We also abstracted induction time (the interval between sedation administration and start of iPFT), procedure time (the interval between iPFT start and completion), and recovery time (the interval between post‐anesthesia care unit entry and discharge). Each study (separately for spirometry and plethysmography) was reviewed for quality by the pediatric pulmonologist and respiratory therapist and categorized as satisfactory or unsatisfactory based on the acceptability and reproducibility. There were no missing data for our variables of interest for this study.

### 2.1 Statistical Analysis

For all analyses, KM, DX, or PP were compared to CH. Bivariate analyses were conducted using Wilcoxon Rank Sum test for continuous variables and Fisher Exact Test for categorical variables. Procedure and total times were stratified by total number of tests attempted, given the inherent impact of number of tests on procedure duration. Multivariable analysis of sedation time, procedure time, recovery time, or total time was conducted using linear regression. Because medications were dosed based on weight, all models were adjusted for weight as well as age. Because the number of tests attempted impacted procedure duration, models of procedure time and total time were additionally adjusted for if MBW was attempted and if BDR was attempted. Analyses were conducted using SAS version 9.4 (SAS Institute, Cary, North Carolina) or R Studio version 4.4.0.

## 3 Results

During the study period, a total of 66 children underwent iPFT. The indications for testing in our patient population were cystic fibrosis (*n* = 54), bronchiolitis obliterans syndrome (*n* = 5), childhood interstitial lung disease (*n* = 3), primary ciliary dyskinesia (*n* = 1), chronic lung disease of prematurity (*n* = 1), autosomal recessive polycystic kidney disease (*n* = 1) and Pompe's disease (*n* = 1). Mean sedation medication doses given to study participants were as follows: CH 104.5 ± 8.7 (range = 92.9–133.5) mg/kg, KM 2.8 ± 1.4 (range = 0.6–5.9) mg/kg, and DX 1.2 ± 0.6 (range = 0.5–2.1) mcg/kg.

Table 1 shows the participant characteristics by sedation strategy. The majority of patients received CH (*n* = 42), followed by KM (*n* = 10), PP (*n* = 8), and DX (*n* = 6). Initial choice of strategy was largely driven by availability of CH and whether the patient had previously had inadequate sedation with CH, and secondarily by preferences of the anesthesiologist. Patients who received CH were younger (median 1.3 years) than those who received KM (median 2.6 years, *p* = 0.0004) or PP (median 2.9 years, *p* = 0.0008) and weighed less (median 9.9 kg) than those who received KM (median 12.4 kg, *p* = 0.0004) or PP (median 13.3 kg, *p* = 0.002). Resting respiratory rate did not differ by sedation strategy. The combination of tests conducted did not significantly differ between CH and the other sedation strategies, nor did the proportion of tests with adequate quality.

**Table 1:** Infant pulmonary function testing participant characteristics by sedation strategy.

	Chloral hydrate (CH)	Ketamine + Midazolam (KM)		Dexmedetomidine (DX)		Polypharmacy (PP)	
	*n* = 42	*n* = 10	*p* *	*n* = 6	*p* *	*n* = 8	*p* *
Age (years)†	1.3 [0.7–1.7]	**2.6 [2.1–3.0]**	**0.0004**	1.7 [0.9–2.1]	0.44	**2.9 [2.3–4.1]**	**0.0008**
Weight (kg)†	9.9 [8.4–11.4]	**12.4 [11.8–12.9]**	**0.0004**	10.3 [9–12.1]	0.44	**13.3 [12–15.1]**	**0.002**
Respiratory Rate (breaths/minute)†	30 [28–35]	33 [32–43]	0.08	29.5 [23–38]	0.78	31 [28–38]	0.65
Tests attempted‡	—	—	0.96	—	0.72	—	0.11
RV‐RTC and plethysmography	9 (21.4%)	2 (20.0%)	—	0 (0%)	—	0 (0%)	—
RV‐RTC, plethysmography, and BDR	7 (16.7%)	1 (10.0%)	—	1 (16.7%)	—	3 (37.5%)	—
RV‐RTC, plethysmography, and MBW	16 (38.1%)	5 (50.0%)	—	3 (50.0%)	—	1 (12.5%)	—
RV‐RTC, plethysmography, BDR, and MBW	10 (23.8%)	2 (20.0%)	—	2 (33.3%)	—	4 (50.0%)	—
RV‐RTC adequate quality‡	29/42 (69.1%)	8/10 (80.0%)	0.70	5/6 (83.3%)	0.66	6/8 (75.0%)	1.00
Plethysmograph adequate quality‡	32/42 (76.2%)	6/10 (60.0%)	0.43	5/6 (83.3%)	1.00	5/8 (62.5%)	0.41
BDR adequate quality‡	12/17 (70.6%)	3/3 (100%)	0.54	2/3 (66.7%)	1.00	6/7 (85.7%)	0.63
MBW adequate quality‡	23/26 (88.5%)	5/7 (71.4%)	0.28	5/5 (100%)	1.00	4/5 (80.0%)	0.52

*Note:* Numbers presented are median [IQR] or number (%). Bold values indicate statistically significant at *p* < 0.05.

Abbreviation: RV‐RTC, raised volume‐rapid thoracoabdominal compression; BDR, bronchodilator response; MBW, multiple breath washout.

* *p*‐value compared to CH.

^†^ Wilcoxon Rank Sum Test.

^‡^ Fisher Exact Test.

**Figure 1:**
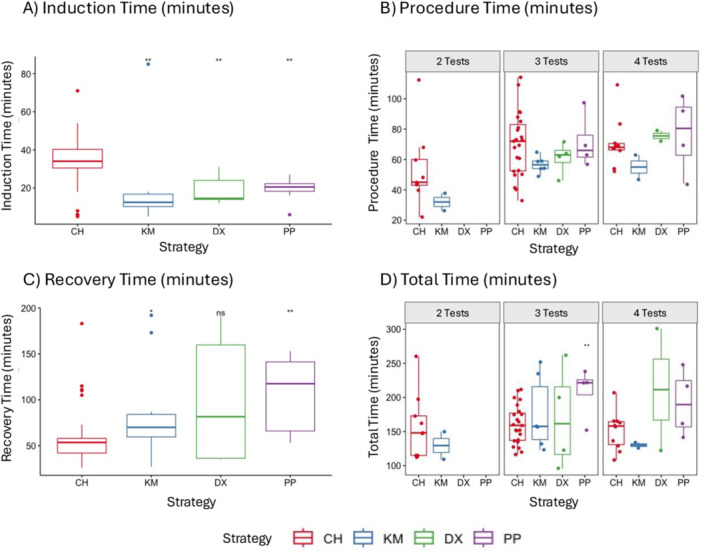
Infant pulmonary function testing times by sedation strategy. Panel (A) shows box plots of the induction time by sedation strategy. Panel (B) shows box plots of the procedure time by sedation strategy and number of tests attempted (since procedure time would be expected to be higher if more tests were attempted). Panel (C) shows box plots of the recovery time by sedation strategy. Panel (D) shows box plots of the total time (induction + procedure + recovery times) by sedation strategy and number of tests attempted (since total time would be expected to be higher if more tests were attempted). CH, chloral hydrate; DX, dexmedetomidine; KM, ketamine + midazolam; ns, not significant. PP, polypharmacy; * indicates *p* < 0.05 compared to CH. ** indicates *p* < 0.01 compared to CH. [Color figure can be viewed at wileyonlinelibrary.com]

Sedation, procedure, recovery, and total times are shown by anesthesia strategy in Figure 1. Procedure and total times were further stratified by total number of tests attempted, given the direct impact of number of tests on procedure duration. Induction times (Panel A) were significantly longer for CH (median duration 34 [IQR 30–41] minutes) compared to each of the other three sedation strategies (median duration 12.5–20.5 min). Compared to CH, procedure times did not significantly differ by sedation strategy among participants who had two, three, or four tests attempted (Panel B, range 22–114 min). Recovery times (Panel C) were significantly shorter for CH (median 53.5 [IQR 42–58] minutes) compared to KM (median 70 [IQR 59–87] minutes) and to PP (median 117.5 [IQR 62–144.5] minutes). Recovery time for DX was also longer than for CH, but the difference was not statistically significant. Compared to CH, for the group that had three tests attempted, total testing time was significantly longer for the PP group (median 221.5 [IQR 186.5–230] minutes) compared to the CH group (median 159 [IQR 136–179] minutes) but did not differ by the other sedation strategies. Compared to CH, total testing time did not differ by sedation strategy among those who had two or four tests attempted (Panel D; the ranges were 109–260 min for two tests attempted and 108–301 min for four tests attempted).

The results for the multivariable analysis of sedation strategy and induction time, procedure time, recovery time, and total time adjusted for weight and age are shown in Table 2. Models of procedure time and total time were additionally adjusted for if MBW was attempted and if BDR was attempted (i.e., number of tests attempted). KM had a shorter procedure time (*β* = −21.9 min, 95% confidence interval [CI] = −38.6 to −5.1, *p* = 0.01) compared to CH; however, total time did not significantly differ. DX had a shorter induction time (*β* = −6.2 min, 95% CI = −11.8 to −0.6, *p* = 0.03) and longer recovery time (*β* = 19.8 min, 95% CI = 3.4–36.2, *p* = 0.02) compared to CH; however, total time did not significantly differ. For PP, recovery time (*β* = 12.0 min, 95% CI = 1.5–22.4, *p* = 0.03) and total time (*β* = 12.0 min, 95% CI = 0.1–23.9, *p* = 0.047) were longer compared to CH. The unadjusted results are presented in E‐Table 1.

**Table 2:** Adjusted analysis of sedation strategy and duration of infant pulmonary function procedures.

Outcome	Beta	(95% Confidence interval)	*p*
**Ketamine + Midazolam (*n*** = **10)**			
Induction Time (minutes)	−11.1	(−24.9 to 2.7)	0.11
Procedure Time (minutes)	**−21.9**	**(−38.6 to −5.1)**	**0.01**
Recovery Time (minutes)	19.0	(−9.9 to 47.9)	0.19
Total Time (minutes)	−8.1	(−39.4 to 23.3)	0.61
**Dexmedetomidine (*n*** = **6)**			
Induction Time (minutes)	**−6.2**	**(−11.8 to −0.6)**	**0.03**
Procedure Time (minutes)	−3.8	(−12.8 to 5.2)	0.39
Recovery Time (minutes)	**19.8**	**(3.4–36.2)**	**0.02**
Total Time (minutes)	11.8	(−7.3 to 31.0)	0.22
**Polypharmacy (*n*** = **8)**			
Induction Time (minutes)	−2.2	(−6.3 to 1.9)	0.29
Procedure Time (minutes)	−1.2	(−8.4 to 6.1)	0.75
Recovery Time (minutes)	**12.0**	**(1.5–22.4)**	**0.03**
Total Time (minutes)	**12.0**	**(0.1–23.9)**	**0.047**

*Note:* Results of the linear regression analysis of sedation strategy and infant pulmonary function testing times. Each sedation strategy was compared to Chloral Hydrate (*n* = 42). All models were adjusted for patient weight and age. Models of procedure time and total time were additionally adjusted for if multiple breath washout was attempted and if bronchodilator response was attempted. Bold values indicate statistically significant at *p* < 0.05.

Adverse events occurred in three patients, two receiving PP and one receiving DX. Within the PP group, one patient experienced hypotension treated with vasopressor and another had a prolonged apneic pause following inflations that lasted 60 s and required positive pressure mask ventilation. A patient in the DX group experienced self‐resolving hypoxemia in the recovery unit. All patients were discharged on the day of their procedures with no subsequent concerns.

## 4 Discussion

Sedation strategies for infant pulmonary function testing in the era of CH unavailability are variable and often guided by individual practice styles, as evidenced by the variety of strategies employed within our institution itself. To our knowledge, our study is the first to compare alternative iPFT sedation strategies to CH. Our findings provide preliminary evidence that KM, DX, and PP may be safe, effective alternatives to CH for iPFT sedation.

In our study, using a KM‐driven strategy showed benefit in reducing procedure time compared to CH without adversely impacting test quality. However, total time did not significant differ between the two strategies. Ketamine is considered a dissociative sedative that binds the N‐methyl‐D‐aspartate (NMDA) receptor. The clinical effect of ketamine sedation produces a trance‐like state with analgesia, amnesia, and immobilization. Importantly, ketamine generally preserves spontaneous breathing, upper airway muscle tone, airway protective reflexes at clinically recommended doses, suggesting the KM approach as a safe CH alternative [14, 15, 16, 17]. The onset of action with ketamine is rapid, within 30 s after intravenous administration, which, in our study, was associated with shortened induction time in the unadjusted analysis.

Our retrospective analysis demonstrated that a DX‐driven strategy slightly reduced induction time and increased recovery compared to CH in adjusted analyses. Dexmedetomidine is a selective alpha‐2 adrenergic receptor agonist that offers a mild, arousable sedation (with stimulation) with minimal respiratory depression in children. Like ketamine, dexmedetomidine preserves upper airway tone, which suggests sedation properties that would be suitable for iPFT similar to CH [18, 19, 20]. Despite all the favorable attributes of dexmedetomidine, it has a relatively long terminal half‐life of 1.6 h in children aged 2–11 years old [21], likely contributing to the increased recovery time on our study.

Our results additionally showed that a polypharmacy‐driven strategy was associated with significantly increased recovery and total time, even after adjusting for age, weight, and number of procedures. Notably, the polypharmacy resulted in two patients that experienced adverse events. The polypharmacy group typically involved the addition of propofol to the sedation approach, and in most instances when the initial sedation approach was failing. Not surprisingly, salvaging the iPFT with propofol after the additional administration of other sedative drugs may have led to the unwanted effects of apnea, prolonged recovery, and/or desaturation events.

Importantly, our study demonstrates no significant differences in the frequency of successful testing when employing anesthesia strategies other than the standard CH. While our results should be interpreted with caution given our sample size, our study suggests that KM and DX can be used in lieu of CH for sedation during iPFT without adversely impacting quality or testing duration. We found no significant differences in the proportion of tests with adequate quality when employing sedation strategies other than the standard CH. For KM and DX, times for some components of iPFTs (e.g., sedation, procedure, or recovery times) differed compared to CH, but importantly, total time did not significantly differ in the adjusted analyses. While we found an increased recovery time and total time for PP, this difference (12.0 min on average in adjusted analyses) may not be clinically meaningful, given that iPFT generally takes a few hours from start to finish. Alternative sedation strategies for iPFTs are being employed globally out of necessity; our study provides preliminary evidence that KM, DX, and PP are non‐inferior alternatives.

This variability in sedation strategy demonstrated in our study is reflected in the broader national landscape of sedation practices for iPFT. Hegde reported on the use of intranasal midazolam monotherapy for iPFTs, noting that the sedative effect was variable despite using maximal dosing [22]. Hayes and colleagues describes the use of propofol with and ketamine, a strategy that most aligns with our PP group, to conduct pulmonary function testing, imaging, and bronchoscopy/BAL/transbronchial biopsy in infant lung transplant recipients [23]. Of the 24 procedural sedations included in their study, one infant required overnight admission due to fever. Our group has previously reported success using dexmedetomidine in a small number of patients (*n* = 4) for infant pulmonary function testing; in that observational study, all attempted iPFT measurements were successfully obtained for all 4 infants receiving dexmedetomidine [24]. Li and colleagues used intranasal Dexmedetomidine in 68 infants sedated for infant pulmonary function testing with similar induction times (onset 12–19 min) to our patient population and with no adverse events in their patient population [25]. However, to the best of our knowledge, our study is the first study to compare these anesthetic strategies for iPFT to CH.

Of note, it is unknown if the existing iPFT reference values for full term healthy controls, which were obtained using CH as the sedation protocol [4, 5], can be applied to subjects who completed iPFT using alternative sedation protocols [3]. Because there are currently no alternative iPFT reference values available for use, we used the current iPFT reference values even for tests conducted with alternative sedation protocols. Determining optimal reference values for alternative iPFT sedation strategies will require future study, which will ideally involve conducting iPFT testing in healthy controls using these alternative sedation protocols and comparing results to the tests conducted using CH.

### 4.1 Limitations

We acknowledge several limitations. Because this was a pilot study, our sample size was small, particularly for each of the three alternative sedation strategies for iPFT. We estimate we would need 65–100 participants per sedation strategy to have adequate power to test for non‐inferiority in our outcomes of interest. As with any observational study, bias is possible, and the sedation strategy (CH vs. alternative) was largely driven by availability of CH and preference of anesthesiologist rather than any patient characteristics. Most participants in our study underwent infant pulmonary function testing for cystic fibrosis, so it is unclear how generalizable our findings are to other disease processes; however, cystic fibrosis is the most common indication for iPFT globally [26]. Additionally, we were unable to adjust for medication doses in our models, given that dose ranges differ by medication type and our small sample size. Moreover, age might be an important covariate as a determinant iPFT success, as obtaining an apnea or brief respiratory pause is critical for obtaining acceptable testing with some methodologies, but we were unable to reliably conduct adjusted analysis of sedation strategy and iPFT success due to small sample size. Future studies with a larger sample size and involving multiple centers should be conducted to validate our findings.

## 5 Conclusions

In light of waning availability of CH, alternative sedation medications are needed to conduct infant pulmonary function testing. We found very preliminary evidence that KM, DX, and PP may be non‐inferior alternatives to CH for iPFT sedation, as the proportion of iPFT that was successful did not differ when employing anesthesia strategies other than the standard CH. However, our pilot results must be interpreted with caution given the very small sample size and observational nature of our study. There may be an increased frequency of adverse events with PP compared to other strategies, but this will require further evaluation with larger sample sizes. Multicenter studies are needed for validation and replication of our findings, but this report provides preliminary evidence that these alternative sedation approaches may be successful and safe options for iPFT in light of waning CH availability.

## Author Contributions

Dr. Aditi Zaveri participated in the conceptualization and design of the study, data entry, data analysis and interpretation, drafting the initial manuscript (lead), and critically reviewing and revising the manuscript. Brian Yoho participated in the conceptualization and design of the study, data collection, and critically reviewing and revising the manuscript. Dr. Brian Blasiole participated in the conceptualization and design of the study, data collection, data interpretation, and critically reviewing and revising the manuscript. Dr. Erick Forno participated in participated in the conceptualization and design of the study, data analysis and interpretation, and critically reviewing and revising the manuscript. Dr. Daniel Weiner participated in the conceptualization and design of the study (lead), data collection, data entry, data analysis and interpretation, drafting the initial manuscript, and critically reviewing and revising the manuscript. Dr. Kristina Gaietto participated in the conceptualization and design of the study, data analysis and interpretation (lead), drafting the initial manuscript, and critically reviewing and revising the manuscript. All authors approved the final manuscript as submitted and agree to be accountable for all aspects of the work.

## Ethics Statement

This is approved by the Institutional Review Board at the University of Pittsburgh (protocol STUDY22010021).

## Conflicts of Interest

The authors declare no conflicts of interest.

## Supporting information


**E‐table 1**. Unadjusted analysis of sedation strategy (compared to Chloral Hydrate, n = 42) and infant pulmonary function testing induction time, procedure time, recovery time, or total time.

## Data Availability

The data that support the findings of this study are available on request from the corresponding author. The data are not publicly available due to privacy or ethical restrictions.
