# Supplementary material for: Impact of Anesthesia Strategy on Infant Pulmonary Function Test Quality and Duration
Source: Pediatr Pulmonol. 2026 Jan 22;61(1):e71477. doi: 10.1002/ppul.71477 (PMC12824825; doi:10.1002/ppul.71477)
Supplement: Supplementary file 1 — E‐table 1. Unadjusted analysis of sedation strategy (compared to Chloral Hydrate, n = 42) and infant pulmonary function testing induction time, procedure time, recovery time, or total time. [file PPUL-61-0-s001.docx]

**Online Supplement**

**Impact of anesthesia strategy on infant pulmonary function test quality and duration**

**Authors:** Aditi K. Zaveri, MD^1^, Brian Yoho, CRT, RPFT^1^, Brian Blasiole, MD, PhD^2^, Erick Forno, MD, MPH^3^, Daniel J. Weiner, MD^†1^, Kristina Gaietto, MD, MPH^†1^

*^1^ Division of Pulmonary Medicine, Department of Pediatrics, UPMC Children’s Hospital of Pittsburgh, Pittsburgh, PA, USA. ^2^ Department of Anesthesia and Perioperative Medicine, UPMC Children’s Hospital of Pittsburgh, Pittsburgh, PA, USA. ^3^ Division of Pulmonology, Allergy/Immunology, and Sleep Medicine, Department of Pediatrics, Riley Hospital for Children, Indianapolis, IN, USA.*

**^†^** Denotes shared senior author.

**Supplementary Methods**

*Functional Residual Capacity-Plethysmography Acceptability Criteria*

Criteria for technical acceptability of functional residual capacity- plethysmography results^1^:

1. Stable end-expiratory level (EEL): variability (SD) of EEL of the last 5 breaths prior to occlusion <5(10)% of mean Vt of these breaths
2. No evidence of leak during occlusion: no decay of pressure plateau during end-inspiratory pause; no flow through the PNT during occlusion; no significant change in EEL after release of occlusion [difference in EEL prior to an after occlusion <15(25)% of the mean Vt]
3. At least two complete respiratory efforts against the occlusion: essential for adequate correction of the drift of Vpleth during airway occlusion
4. No phase lag or looping between Vpleth and Pao; no signs of glottic closure or permanent or intermittent airway obstruction precluding rapid and adequate equilibration of alveolar mouth pressures (x-y plot at appropriate magnification with a minimal angle of ~30 degrees recommended to ascertain such signal quality)
5. Reported results based on 3 acceptable occlusions: mean, SD, and/or coefficient of variation to be reported. CV <10% between results from the 3 acceptable occlusions.
6. Cross-check of results by an independent observer if possible.

*Raised Volume-Rapid Thoracoabdominal Compression Acceptability Criteria*

Technically acceptable maneuvers for raised volume-rapid thoracoabdominal compression (RV-RTC)^2^ are those in which:

1. Relaxation of the respiratory system is evident before the RTC maneuver (i.e., passive expiration, confirmed by visual inspection of the volume–time plot showing an exponential decay of flow) and where forced expiration proceeds smoothly without evidence of early inspiration, marked flow transients, or glottic closure.
2. In addition, peak expiratory flow should be achieved before 10% volume is expired to avoid reporting of supramaximal flows at low lung volumes due to late or slow application of the forcing function.
3. If errors are to be avoided in assessments of FVC and FEF at fixed proportions of FVC (FEF%), it is essential to ensure the infant has breathed out fully toward RV, ideally with a brief apneic pause after release of the jacket pressure.
4. At least two technically acceptable maneuvers are required before reporting a result, provided that FVC, FEF25–75, and FEV0.4/0.5 are within 10% of each other. If there is only one good curve or variability is greater than 10% between the two maneuvers, then no result should be reported.
5. The commonly reported parameters calculated from the RVRTC are as follows: FVC, FEV0.5, FEF50, FEF75, FEF85, and FEF25–75. In infants younger than 3 months, FEV0.4 should also be reported because rapid lung emptying at this age often precludes measurements made beyond 0.5 s (15, 27). For clarity, FEF% (U.S. convention) defines the forced expiratory flow when %FVC had been expired while MEF% (European convention) relates to forced flows when %FVC remains in the lungs (i.e., FEF75 is equivalent to MEF25).
6. While maximal flow at FRC (V˙ maxFRC) is the parameter of primary interest obtained from partial forced expiratory maneuvers in infants, this parameter is not reported from RVRTC because the “end expiratory level” during the period of lung inflations may either be lower than that maintained dynamically during spontaneous breathing due to complete passive expiration, or relatively elevated due to presence of inadvertent or deliberate positive end-expiratory pressure. For this reason, reporting V˙maxFRC from RVRTC is likely to be highly variable and would not correspond to that obtained from partial curves.
7. Forced flows and volumes should be reported from the “best” trial. The best trial is generally defined as the one with either the highest sum of FVC and FEV0.4/0.5 (15, 21) or the highest sum of FVC and FEF25–75 (19, 40). Differences between these two methods of selecting the “best” loop have been reported to be minimal (41). In contrast to partial forced expiratory maneuvers, these measurements are independent of changes in FRC, and it is therefore recommended that values from the “best” curve (not mean) are reported. The three best curves should be used for the calculation of mean (SD) and coefficient of variation, where “best” is based on technical criteria.

*Bronchodilator Responsiveness Acceptability Criteria and Protocol*

Criteria for technical acceptability of bronchodilator responsiveness results were the same as for RV-RTC testing.

Bronchodilator responsiveness was tested by administering 2 puffs of albuterol every 2 minutes until a maximum dose of 8 puffs or 20% change in heart rate, as per previous guidelines^3^.

*Multiple Breath Washout Acceptability Criteria*

Criteria for technical acceptability of multiple breath washout results^4^:

1. Stable VT for 10 breaths/30 seconds prior to wash in as seen on CO2 trace
2. Gas equilibrium variability <1% of each other (0.002% if 0.2% gas used)
3. A further 5-10 breaths over 20-30seconds
4. Regular breathing pattern for the first 10 breaths of wash out

**Supplementary Tables**

**E-table 1.** Unadjusted analysis of sedation strategy (compared to Chloral Hydrate, n=42) and infant pulmonary function testing induction time, procedure time, recovery time, or total time.

| **Outcome** | **Beta** | **(95% Confidence Interval)** | **p-value** |
| --- | --- | --- | --- |
| **Ketamine + Midazolam (n=10)** | | | |
| Induction Time (minutes) | **-13.9** | **(-25.1, -2.8)** | **0.02** |
| Procedure Time (minutes) | **-15.2** | **(-29.5, -0.9)** | **0.04** |
| Recovery Time (minutes) | **29.0** | **(-4.7, 53.4)** | **0.02** |
| Total Time (minutes) | -0.1 | (-25.5, 25.3) | 0.99 |
| **Dexmedetomidine (n=6)** | | | |
| Induction Time (minutes) | **-7.2** | **(-12.9, -1.5)** | **0.02** |
| Procedure Time (minutes) | -0.4 | (-9.5, 8.7) | 0.93 |
| Recovery Time (minutes) | **20.7** | **(4.8, 36.6)** | **0.01** |
| Total Time (minutes) | 13.1 | (-5.1, 31.4) | 0.15 |
| **Polypharmacy (n=8)** | | | |
| Induction Time (minutes) | **-4.7** | **(-7.9, -1.4)** | **0.007** |
| Procedure Time (minutes) | 2.5 | (-3.1, 8.1) | 0.37 |
| Recovery Time (minutes) | **16.3** | **(8.2, 24.4)** | **0.0002** |
| Total Time (minutes) | **14.1** | **(5.3, 23.0)** | **0.002** |
| Linear regression results for sedation strategy and infant pulmonary function testing times. For each model, the sedation strategy was compared to Chloral Hydrate (n=42). | | | |

**References:**

1. ATS/ERS statement: raised volume forced expirations in infants: guidelines for current practice. *Am J Respir Crit Care Med.* 2005;172(11):1463-1471.

2. Stocks J, Godfrey S, Beardsmore C, Bar-Yishay E, Castile R. Plethysmographic measurements of lung volume and airway resistance. ERS/ATS Task Force on Standards for Infant Respiratory Function Testing. European Respiratory Society/ American Thoracic Society. *Eur Respir J.* 2001;17(2):302-312.

3. Goldstein AB, Castile RG, Davis SD, et al. Bronchodilator responsiveness in normal infants and young children. *Am J Respir Crit Care Med.* 2001;164(3):447-454.

4. Shawcross A, Murray CS, Pike K, Horsley A. A novel method for infant multiple breath washout: First report in clinical practice. *Pediatric Pulmonology.* 2019;54(8):1284-1290.
